# Metabolic multistability and hysteresis in a model aerobe-anaerobe microbiome community

**DOI:** 10.1126/sciadv.aba0353

**Published:** 2020-08-12

**Authors:** Tahmineh Khazaei, Rory L. Williams, Said R. Bogatyrev, John C. Doyle, Christopher S. Henry, Rustem F. Ismagilov

**Affiliations:** 1 Division of Biology and Biological Engineering, California Institute of Technology, Pasadena, CA, USA.; 2 Division of Engineering and Applied Science, California Institute of Technology, Pasadena, CA, USA.; 3 Data Science and Learning Division, Argonne National Laboratory, Lemont, IL, USA.; 4 Division of Chemistry and Chemical Engineering, California Institute of Technology, Pasadena, CA, USA.

## Abstract

Major changes in the microbiome are associated with health and disease. Some microbiome states persist despite seemingly unfavorable conditions, such as the proliferation of aerobe-anaerobe communities in oxygen-exposed environments in wound infections or small intestinal bacterial overgrowth. Mechanisms underlying transitions into and persistence of these states remain unclear. Using two microbial taxa relevant to the human microbiome, we combine genome-scale mathematical modeling, bioreactor experiments, transcriptomics, and dynamical systems theory to show that multistability and hysteresis (MSH) is a mechanism describing the shift from an aerobe-dominated state to a resilient, paradoxically persistent aerobe-anaerobe state. We examine the impact of changing oxygen and nutrient regimes and identify changes in metabolism and gene expression that lead to MSH and associated multi-stable states. In such systems, conceptual causation-correlation connections break and MSH must be used for analysis. Using MSH to analyze microbiome dynamics will improve our conceptual understanding of stability of microbiome states and transitions between states.

## Teaser

Multistability and hysteresis is a potential mechanism to describe shifts to and persistence of aerobe-anaerobe communities.

## INTRODUCTION

Recent evidence shows that changes in the species composition and abundance of the human microbiome can be associated with health and disease (*1*–*3*). Understanding the mechanisms that cause compositional shifts in healthy microbiomes, which otherwise can be stable, is challenging because of the inherent complexity of these ecosystems. A perplexing feature of some of these disturbed ecosystems is the persistence of a new microbiome state, even in seemingly unfavorable conditions. For example, in small intestinal bacterial overgrowth (SIBO), strict anaerobes that are typically found only in the colon become prominent in the small intestine and, paradoxically, persist in this environment exposed to oxygen flux from the tissue (*4*, *5*). Similarly, in periodontal diseases (*6*) and in wound infections, anaerobes proliferate in oxygen-exposed environments. 

**Fig. 1:**
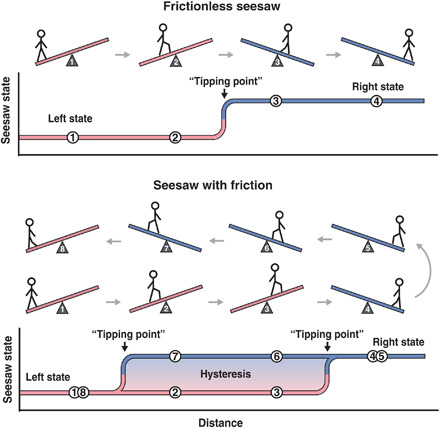
Simplified illustration of multistability and hysteresis. A frictionless seesaw is a multistable system with two states (left and right) determined by the position of the person. Conversely, a seesaw with static friction is a multistable system with hysteresis. Within the region of hysteresis, the state of the system is not determined by the external input (position of person) but rather by the system’s history. As the person walks through positions 1 and 2 past the midpoint to position 3, the seesaw remains stuck in the left state; the person has to walk much further, to position 4, to switch it to the right state. As the person walks back to the left, through positions 5 and 6 and past the midpoint to position 7, the seesaw remains stuck in the right state and it only switches back to the left state when the person reaches position 8. In the region of positions 2, 3, 6, and 7, the system exhibits multistability and hysteresis (MSH). Here, the state of the microbial community (*Kp*-only state or *Kp-Bt* state) is analogous to the state of the seesaw, and the balance of oxygen or glucose inputs are analogous to the positions of the person.

One potential mechanism to explain microbiome shifts and their persistence is multistability (*7*–*13*), the concept that several steady states can exist for an identical set of system parameters (Fig. 1). Multistable systems have been described in the context of ecosystems (*14*–*17*) and gene regulatory networks (*18*–*20*). Now, with the expanding characterization of the microbiome, there are signs that multistability may also exist in these communities (*21*–*27*). For example, compositional changes in gut microbiota are implicated in inflammatory bowel disease (*28*) and obesity (*29*). Bimodal species abundance (i.e., when a microbial species is present at either high or low levels) has been interpreted as multistability (*30*); however, as discussed by Gonze *et al.*, bimodality is insufficient to prove multistability (*7*, *31*). Some multistable systems can additionally exhibit hysteresis, where in response to a perturbation, a system gets “stuck” in a new steady state and the former state cannot be regained by simply reversing the perturbation (*7*). The presence of hysteresis could be hypothesized from studies of the microbiome (*32*). For example, antibiotic exposures can change the microbiome composition and have lasting effects even after removal of the antibiotic (*33*, *34*). However, it has not been rigorously tested whether multistability and hysteresis (MSH) can arise in a microbiome-relevant community and by what mechanism.

Here, we investigate MSH in a minimally “complex” two-species system to represent the paradoxical aerobe-anaerobe microbiome communities that persist in oxygen-exposed environments. We used two organisms prevalent in SIBO (*35*): the anaerobe *Bacteroides thetaiotaomicron* (*Bt*) that breaks down complex carbohydrates (e.g., dextran) into simple sugars and short-chain fatty acids (*36*) and the facultative anaerobe (hereafter referred to as an aerobe) *Klebsiella pneumoniae* (*Kp*) capable of consuming oxygen, simple sugars, and short-chain fatty acids and performing anaerobic respiration in the absence of oxygen (*37*).

**Fig. 2:**
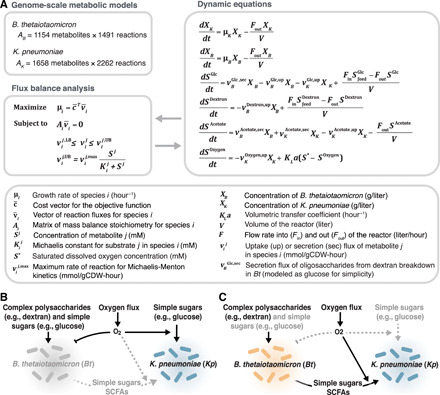
A multistable model system consisting of *Kp*, a facultative anaerobe, and *Bt*, an anaerobe, that is relevant to the human gut microbiome. (**A**) Dynamic equations describing the model system can be solved with dFBA using each species’ genome-scale metabolic model. (**B**) In the *Kp-*only state, *Bt* does not grow and *Kp* uses external sugars and short-chain fatty acids. (**C**) In the *Kp-Bt* state, *Bt* can grow and break down complex polysaccharides into simple sugars and short-chain fatty acids, which *Kp* can use to maintain reduced oxygen levels favorable for *Bt* growth.

Mathematical simulations (Fig. 2A) and a 35-day (832 hours) experiment (fig. S1) in a continuously stirred tank reactor (CSTR) revealed that MSH occurs in our two-species system with two distinct steady states that can exist under identical environmental conditions: (i) *Kp*-only state and (ii) *Kp-Bt* state, where the anaerobe (*Bt*) proliferates in the presence of continuous oxygen input. Using genome-scale mathematical models, which capture the full metabolic capacity of each species (1491 equations for *Bt* and 2262 equations for *Kp*), and RNA sequencing data collected from the CSTR experiment, we find that MSH extends to the level of metabolism, where genes are differentially expressed in the two distinct states. We identified key metabolic pathways (short-chain fatty acid and oligosaccharide metabolism) involved in metabolic coupling between the two species leading to MSH.

## RESULTS

### Computational results

Mathematical simulations of a CSTR revealed that MSH arises from the interplay between environmental perturbations and interspecies metabolic interactions. We used the dynamic multispecies metabolic modeling (DMMM) framework (*38*) to model a community of *Kp* and *Bt* in a CSTR (Fig. 2A) with continuous input flows of dextran minimal media and varying input glucose or varying input oxygen levels (depending on the simulation). The outflow rate is equal to the inflow rate to maintain a constant reactor volume, and the resident time in the reactor is 5 hours. The DMMM framework uses dynamic flux balance analysis (dFBA) (*39*), which allows us to capture temporal changes in intracellular flux rates (using the genome-scale metabolic model for each species), extracellular metabolite concentrations, and species concentrations.

**Fig. 3:**
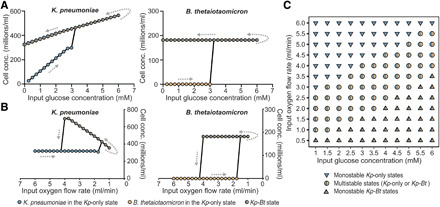
Simulations illustrating MSH in the microbial community with respect to environmental perturbations. Cell concentrations as a factor of (**A**) glucose concentration variations in the input feed under constant input oxygen flow rate (1.7 ml/min) and (**B**) input oxygen flow variations under constant glucose concentrations (3 mM) in the input feed. Each point represents the steady-state concentration for the given species in the community after a 50-hour simulation. (**C**) Regions of stability as a function of glucose concentrations in the input feed and oxygen flow rates into the reactor. In regions of multistability (circles), the community can exist in either a *Kp*-only state or a *Kp-Bt* (aerobe-anaerobe) state under the same conditions. In regions of monostability (triangles), it is only possible for one state to exist for the given set of parameters; down-pointed triangles represent monostable regions where *Kp*-only state exists, and up-pointed triangles represent monostable regions where only the *Kp-Bt* state can exist.

To computationally test whether a change in the balance of oxygen and carbon fluxes could lead to a change in the state of the aerobe-anaerobe community, we altered glucose input concentrations (Fig. 3A), while keeping constant all other system parameters, including continuous oxygen input and continuous dextran input. The model predicted that for glucose concentrations of 0.25 to 3 mM in the input feed (at a constant flow rate of 0.7 ml/min for all conditions), the output state consisted solely of *Kp*, which we refer to as the *Kp*-only state (Fig. 2B). Stoichiometrically, at these glucose concentrations, oxygen was not completely consumed; thus, the environment was unfavorable for *Bt* growth. However, when we increased glucose input concentration to 3.25 mM, we observed a shift to a new steady state (Fig. 3A). At this “tipping point,” the environment became sufficiently anaerobic to support the growth of *Bt*. We refer to this second distinct steady state as the *Kp-Bt* (aerobe-anaerobe) state (Fig. 2C). In the *Kp-Bt* state, the *Kp* population is no longer carbon limited because of the additional carbon sources generated from the metabolism of dextran by *Bt*. Thus, *Kp* can now consume all of the available oxygen to oxidize both glucose and the additional carbon sources, resulting in anaerobic conditions. Unexpectedly, this *Kp-Bt* state persisted when we systematically reversed the input of glucose below 3.25 mM, even to 0 mM. Thus, this system shows hysteresis and multistability: Under identical input conditions of glucose and oxygen, the system can be in either of the two possible states. We then identified tipping points for population shifts in response to input oxygen variations, with glucose kept constant (Fig. 3B). In addition to glucose input, all other parameters, including continuous dextran input, were held constant. We considered oxygen as a parameter because in host settings, oxygen availability can be affected by respiration, blood flow rate, immune consumption, etc. We found that we could return the system to the *Kp*-only state by increasing oxygen levels, a state switch that was not possible by manipulating glucose concentration alone. Last, we simulated changes in both glucose and oxygen levels and characterized the landscape of multistability and monostability in the model microbial community (Fig. 3C). These simulation results illustrate that even a minimal model of microbiome with codependence (*40*, *41*) can demonstrate marked MSH.

### Experimental results

To confirm and further explore MSH beyond mathematical predictions, we performed a CSTR experiment over 35 days (832 hours). In the CSTR (200-ml culture volume), we varied input glucose concentrations (while keeping all other parameters constant, including continuous dextran and oxygen) and measured the steady-state output composition of the microbial community by quantitative polymerase chain reaction [qPCR; and digital PCR (dPCR); fig. S6]. Oxygen was sparged into the reactor at 3.4% of the gas feed (total gas feed, 50 ml/min) and kept constant for all conditions. For each steady-state condition, we collected three CSTR samples separated by at least one residence time (5 hours; fig. S1 contains the experimental workflow).

**Fig. 4:**
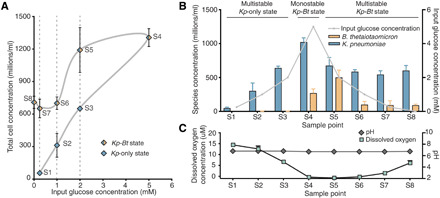
MSH of *Kp* and *Bt* community in a CSTR. (**A**) Total cell concentrations collected at the eight different steady-state sample points (S1 to S8) from the CSTR measured by qPCR. (**B**) Cell concentrations for each individual species in the community measured by qPCR. (**C**) pH and dissolved oxygen concentrations measured in the CSTR for each sample point. Error bars are SD of at least three replicates collected at steady state (separated by >1 residence time of 5 hours) from the CSTR for each of the eight steady-state glucose conditions. See fig. S1 for bioreactor workflow.

As predicted by the mathematical models, we observed both multistability and hysteresis (Fig. 4A) experimentally. At 0.25, 1, and 2 mM glucose concentrations, the steady-state community consisted only of *Kp*; *Bt* was washed out under these conditions (Fig. 4B). To confirm washout, *Bt* was reinoculated three separate times. The dissolved oxygen measurements (Fig. 4C) confirmed that oxygen was not limiting under the selected parameter conditions, resulting in an aerobic environment unsuitable for *Bt* growth. As in the simulations, at 5 mM glucose, a new distinct steady state was reached where *Bt* grew in the presence of *Kp.* Although there was continuous oxygen flux into the reactor, the concentration of dissolved oxygen measured in the reactor was near zero. Next, to test for hysteresis, we reduced the glucose input back down to 2, 1, 0.25, and 0 mM and found that the aerobe-anaerobe state persisted. The persistence of the *Kp-Bt* state (instead of a return to the *Kp*-only state) qualitatively confirmed model predictions of hysteresis and verified that this microbial community is a multistable system.

The CSTR results demonstrate metabolic coupling and codependence between these two bacterial species with respect to carbon and oxygen. At sample point 8, there is no glucose input to the reactor, yet *Kp* continued to grow, indicating that *Kp* was completely dependent on *Bt* for its carbon supply. At sample point 4, *Bt* started to grow, despite the continuous oxygen input, indicating that *Bt* was dependent on removal of oxygen by *Kp*. At sample points 7 (0.25 mM glucose) and 8 (0 mM glucose), *Bt* continued to grow, despite dissolved oxygen measurements indicating oxygen concentrations above the tolerance for *Bt* growth (Fig. 4C). This observation differed slightly from the model, suggesting that there may be additional biological factors beyond metabolic coupling and stoichiometric balance of carbon and oxygen that can affect multistability. Imaging revealed that in the *Kp-Bt* state, bacterial aggregates were larger at lower glucose concentrations. Furthermore, fluorescent in situ hybridization showed these aggregates contained both *Kp* and *Bt* (fig. S2). We hypothesize that coaggregation is one potential mechanism that could extend the region of hysteresis by providing microenvironments more favorable for *Bt* growth by further facilitating metabolic coupling between the two species, as observed in biofilms (*6*). Other factors, such as adhesion to the walls of the vessel, may also contribute to extending the region of hysteresis.

**Fig. 5:**
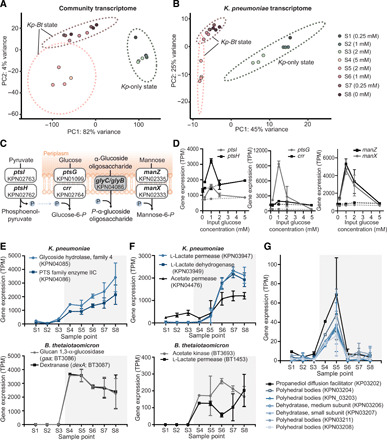
Gene expression analysis of CSTR steady-state samples. (**A**) PCA of the community transcriptome; each point represents the combined transcriptome of *Kp* and *Bt* for each sample (S1 to S8). (**B**) PCA of the *Kp* transcriptome. (**C**) The most differentially regulated pathway between the *Kp-*only and the *Kp-Bt* states is the PTS. The gray box indicates the up-regulated gene; white boxes are down-regulated genes. (**D**) PTS genes down-regulated in the *Kp-Bt* state in *Kp*. Solid lines represent the *Kp-*only state, and dashed lines represent the *Kp-Bt* state. (**E**) Gene expression, in transcripts per million (TPM), of oligosaccharide uptake in *Kp* and dextran metabolism to oligosaccharides in *Bt* for each steady-state sample point. (**F**) Expression of genes involved in acetate and lactate utilization in *Kp* and acetate and lactate production in *Bt* for each CSTR sample. (**G**) Expression of the propanediol utilization pathway in *Kp.* (E to G) Unshaded regions are the *Kp-*only state; the gray shaded region is the *Kp-Bt* state. Error bars are SD of three replicates collected (separated by >1 residence time) from the CSTR for each of the eight steady-state glucose conditions.

Gene expression analysis of CSTR samples revealed that multistability also occurs at the transcriptome level in both the community and in individual species. Principal components analysis (PCA) of the community-level gene expression data showed that samples clustered on the basis of the steady state (*Kp-*only versus *Kp-Bt*) from which they were collected (Fig. 5A). Strong clustering at the community level is expected because *Bt* is absent from the *Kp*-only state. However, when we evaluated the gene expression profile of *Kp* (Fig. 5B), which is present in all steady-state conditions, we also found clustering based on the state of the community.

To further evaluate the proposed metabolic mechanism responsible for MSH (Fig. 2, B and C), we compared metabolic regulation in *Kp* in the *Kp-Bt* state and the *Kp-*only state. We used a method from the Neilsen Lab (*42*) to collect topological information from the genome-scale metabolic models and combine it with gene expression data to identify reporter metabolites that maximally differ between the two states. Among the top reporter metabolites were pyruvate, phosphoenolpyruvate, glucose, and glucose-6-phosphate (table S3), suggesting that the phosphotransferase system (PTS), which is involved in sugar transport, is up-regulated in the *Kp*-only state relative to the *Kp-Bt* state (Fig. 5, C and D). In the *Kp-Bt* state, genes involved in the α-glucoside linked substrates were up-regulated (Fig. 5E), suggesting that *Kp* obtains some of its carbon source from oligosaccharides. These oligosaccharides are released into the environment by *Bt* through the breakdown of dextran by dextranase, an extracellular endohydrolase (*43*). *Bt* uses these oligosaccharides by hydrolyzing them using glucan-1,3-α-glucosidases. As expected, both dextranase (*dexA*) and glucan-1,3-α-glucosidase (*gaa*) were found to be highly expressed in *Bt* in the *Kp-Bt* state (Fig. 5E).

Our analysis (Fig. 5F) also suggested an up-regulation of acetate utilization by *Kp* in the *Kp-Bt* state as inferred from the up-regulation of acetate permease. In addition, *Kp* genes involved in lactate utilization were up-regulated in the *Kp-Bt* state. Upon oxygen exposure, *Bt* is known to produce lactate (*44*). A pilot experiment showed that the addition of a mixture of lactate and acetate can cause a direct “jump” from the *Kp-*only state to the *Kp-Bt* state (fig. S5), emphasizing that short-chain fatty acids are involved in the metabolic coupling between *Kp* and *Bt* in MSH. Multistability of gene expression extended to the anaerobic metabolic pathway for propanediol utilization in *Kp* (Fig. 5G) (*45*). We thus infer that a subpopulation of *Kp* was undergoing anaerobic metabolism in samples 4 and 5 (of the *Kp-Bt* state), where the dissolved oxygen concentrations in the reactor were lowest (Fig. 4C). Overall, these results were consistent with the basic mechanism for MSH (Fig. 2, B and C) and reveal that MSH extends to the expression of genes and pathways involved in metabolic coupling between the species.

## DISCUSSION

In this work, we used genome-scale mathematical modeling, bioreactor experiments, transcriptomics, and dynamical systems theory to show that MSH is a mechanism that can describe shifts and persistence of a two-member model microbiome aerobe-anaerobe community under seemingly paradoxical conditions (e.g., oxygen-exposed environments). We further identified key metabolic pathways involved in MSH in the *Kp-Bt* system. Future gene knockout studies would further confirm the critical metabolic pathways responsible for MSH. We demonstrated that altering the balance between carbon and oxygen fluxes within the system, by changing input glucose levels, leads to a community shift from the *Kp*-only state to the *Kp-Bt* state. Follow-up experiments exploring the manipulation of input oxygen levels would be needed to quantitatively evaluate the role of input oxygen levels in MSH; in particular, the model-predicted ability to switch the system back to the *Kp*-only state. A limitation of this study is the long time frame of the CSTR experiment (fig. S1) and that the data reported come from a single run; however, a shorter pilot experiment (fig. S9) demonstrated similar dynamics. More broadly, identifying and interpreting MSH in human microbiomes and microbiome-associated diseases would require carefully designed longitudinal measurements and models that take into account the full complexity of microbiomes, their spatial structure, and host responses. If MSH is found, then it would have profound conceptual impact. To understand and control microbial communities without MSH, one currently relies on a well-established conceptual connection between correlation, causation, and control. Consider points S1 to S3 (Fig. 4). The levels of *Kp* correlate with the input glucose concentration; from a known input glucose concentration, one can infer a steady-state *Kp* concentration and vice versa. Input glucose concentration is the causal factor, and therefore, it can be used to control the steady-state levels of *Kp*. If MSH is identified in microbiomes, then it would break this familiar conceptual connection between causation and correlation. Consider the region of hysteresis (points S1 to S3 and S5 to S7; Fig. 4). The observed steady-state levels of *Kp* no longer correlate with the input glucose concentration. At 2 mM input glucose, the system could be in either the *Kp*-only state S3 or the *Kp-Bt* state S5. At ~650 × 10^6^ colony-forming units/ml of *Kp*, the input glucose levels could be either 0.25 or 2 mM. Although there is no correlation between species’ abundance and input glucose concentration (in other words, knowing glucose concentration is not sufficient for predicting species abundance; instead, a system’s history must also be known), input glucose concentration remains the causal factor. Furthermore, under MSH, establishing causation is insufficient for achieving control: Although input glucose concentration is the causal factor responsible for changes in the community state, it cannot be used to fully control the community (i.e., one cannot use changes in glucose inputs to revert the *Kp-Bt* state back to the *Kp*-only state). Alternative control strategies (e.g., changes in oxygen levels or disruption of metabolic coupling), derived from appropriate models, would need to be deployed under MSH. Therefore, recognizing whether and when MSH exists in human microbiomes will be critical for interpreting correlation and causation and for designing therapeutic control strategies that can steer microbial communities to desirable states.

## MATERIALS AND METHODS

### Model development

For the computational simulations, we used the DMMM framework (*38*), which is an extension of dFBA applied to microbial communities. Briefly, the DMMM framework has two components. In component 1, external differential equations describe mass balances for species and metabolite concentrations in the CSTR (shown in Fig. 2A and described in the Supplementary Materials). Unlike the traditional method for solving differential equations in a bacterial system, we do not assume that parameters such as growth rates and metabolic flux rates are constant. Instead, we allow the parameters to be dynamic because we are studying a system with potentially rich dynamics. To find the values for these dynamic parameters, we use FBA (component 2) to solve for the parameter values at every time step of the simulated time period. Component 2 includes the genome-scale models for each species. These models are used to perform FBA at every time point to obtain updated parameters for the differential equations in component 1.

#### Component 1

The system is described as a CSTR with the following mathematical formulation

$$\frac{dX_{i}}{\mathrm{dt}}=\mu_{i}X_{i}-\frac{F_{\text{out}}X_{i}}{V}$$ (1)

$$\frac{{\mathrm{dS}}^{j}}{\mathrm{dt}}=\sum_{i}v_{i}^{j}X_{i}+\frac{F_{\text{in}}S_{\text{feed}}^{j}-F_{\text{out}}S^{j}}{V}$$ (2)

$$\frac{{\mathrm{dS}}^{\text{oxygen}}}{\mathrm{dt}}=\sum_{i}v_{i}^{\text{oxygen}}X_{i}+K_{L}a(S*-S^{\text{oxygen}})$$ (3)

Here, *V* is the volume of the reactor (constant), and *X_i_* is the biomass (g/liter) of the *i*th microbial species. *S^j^* is the concentration (mM) of the *j*th metabolite, *F*_in_ is the rate of flow (liter/hour) into the reactor, *F*_out_ is the rate of flow (liter/hour) out of the reactor, $S_{\text{feed}}^{j}$ is the concentration of the *j*th metabolite in the feed stream, μ*_i_* (hour^−1^) is the growth rate of the *i*th microbial species, $v_{i}^{j}$ is the metabolic flux of the *j*th substrate in the *i*th microbial species, *K_L_a* is the volumetric oxygen transfer coefficient, and S* is the dissolved oxygen saturation concentration.

In continuous culture, substrate utilization can deviate from diauxic growth (as typically observed in batch culture) and cosubstrate utilization is possible (*46*). The set of differential equations are solved using the following analytical approximation

$$X_{f}=X_{i,0}e^{(\mu_{i}-\frac{F_{\text{in}}}{V_{0}})∆T}$$ (4)

$$\begin{array}{c} S_{f}^{j}=S_{0}^{j}+\sum_{i}[v_{i}^{j}\frac{V_{0}}{\mu_{i}V_{0}-F_{\text{in}}}(X_{i,0}e^{(\mu_{i}-\frac{F_{\text{in}}}{V_{0}})\Delta T}-X_{i,0})]\\ +\frac{F_{\text{in}}(S_{\text{feed}}^{j}-S^{j})}{V_{0}}\Delta T \end{array}$$ (5)

$$\begin{array}{c} S_{f}^{\text{oxygen}}=S_{0}^{\text{oxygen}}+\sum_{i}[v_{i}^{\text{oxygen}}\frac{V_{0}}{\mu_{i}V_{0}-F_{\text{in}}}(X_{i,0}e^{(\mu_{i}-\frac{F_{\text{in}}}{V_{0}})\Delta T}-X_{i,0})]\\ +K_{L}a(S*-S^{\text{oxygen}})\Delta T \end{array}$$ (6)

At the beginning of every time step (∆*T*), the parameters μ*_i_* and $v_{i}^{j}$ are calculated using FBA from genome-scale models (component 2) and fed back into Eqs. 4, 5, and 6. This process is repeated for all time intervals in the simulated time period.

#### Component 2

Genome-scale metabolic models are used to establish genotype-phenotype relationships and capture the metabolic capabilities of each model organism. Furthermore, these models allow us to integrate metabolic network topology information with RNA sequencing data for transcriptomic analysis of the CSTR experiments (described further in the “RNA sequencing and analysis” section). We used the published iYL1228 model of *Kp* MGH-78578 (*37*) and the published iAH991 model of *Bt* VPI-5482 (*36*). Because these models are well validated (and curated), we added the minimum number of parameters that allow for integration of these models to the community dFBA framework. Our changes include adding a pathway for dextran uptake and hydrolysis to glucose in the *Bt* iAH991 model. The pathway lumps hydrolysis of dextran to glucose into a single reaction. In this lumped reaction, we assume that 50% of the glucose produced from dextran by *Bt* can be released into the environment for shared use. For the purpose of the simulations, dextran is assumed to be 100 glucose units. The genome-scale models are solved separately for each species by FBA (*47*) at each time point

$$\text{max}\;c^{T}\bar{v_{i}}$$ (7)

$$s.t.A_{i}\bar{v_{i}}=0$$

$${\bar{v}}_{i,\text{lb}}<\bar{v_{i}}<{\bar{v}}_{i,\text{ub}}$$

where *c* is the cost vector, $\bar{v}$ is the vector of fluxes, and *A* is the matrix of mass balance stoichiometries. The optimization criterion is biomass growth rate (for each species). For the bounds for the fluxes, we used the values in the curated, published genome-scale models (*Kp* iYL1228 and *Bt* iAH99). The uptake fluxes explicitly modeled in component 1 (dextran, glucose, acetate, and oxygen) are bounded by Michaelis-Menten kinetics

$$v_{i,\text{ub}}^{j}=v_{i}^{j,\text{max}}\frac{S^{j}}{K_{m}+S^{j}}$$ (8)

The values for $v_{i}^{j,\text{max}}$ and *K_m_* for some of the metabolites in the model were estimated from batch experiments. Batch culture experiments were carried out in a 96-well flat-bottom plate. Overnight cultures grown anaerobically in minimal medium supplemented with either 0.5% (w/v) dextran or 0.5% (w/v) glucose were diluted 1:20 (for *Bt*) and 1:100 (for *Kp*) and outgrown to mid-log phase. The cultures were then pelleted and resuspended at optical density (OD) 1 (for *Bt*) and OD 0.1 (for *Kp*) in carbon-source-free minimal medium. We added 10 μl of cells to 200 μl of minimal medium containing various concentrations [0.125 to 0.5% (w/v)] of the carbon source. The plate was incubated at 37°C, and OD_600_ was measured every 10 min. For batch cultures, Monod growth kinetics was assumed

$$\frac{\mathrm{dX}}{\mathrm{dt}}=X\mu_{\text{max}}\frac{S}{S+K}$$ (9)

$$\frac{\mathrm{dS}}{\mathrm{dt}}=-v_{\text{max}}\frac{S}{S+K}$$ (10)

where μ_max_ is the maximum growth rate the given bacteria can achieve on a carbon source when it is not resource limited. Growth data from replicate wells of multiple concentrations of carbon source were fitted simultaneously using Bayesian parameter estimation implemented with Markov chain Monte Carlo (*48*). Individual growth curves were allowed to have distinct initial cell concentrations and background values, with other parameters held constant. The fitted parameters are presented in table S1 and fig. S3. For all other metabolites captured in the differential equations, the *K*_m_ and *v*_max_ values are assumed to be the same (*v*_max_ of 10 mmol/gCDW·hour and *K*_m_ of 0.01 mM) on the basis of literature for *Escherichia coli* (*49*, *50*).

Values for parameters and initial conditions used in the model are presented in table S2. Initial conditions are chosen to represent the experimental setup, whereby we first establish a steady state for *Kp* in the CSTR before inoculating *Bt*. Therefore, in the models, we start with a higher concentration of *Kp* than *Bt*. The initial conditions for *Kp* in the reactor is arbitrarily chosen to be the experimentally measured monoculture steady-state concentration of *Kp* at an input glucose concentration of 0.25 mM. The initial conditions for *Bt* in the reactor are 0.0015 g/liter, which is equivalent to addition of 1 ml of OD 1 *Bt* into the reactor, as done experimentally. For most steady-state conditions, glucose is limiting, and therefore, the initial conditions for glucose concentration in the reactor are chosen to be 0 mM.

We note that although an individual FBA simulation (component 1) is linear, a dynamic FBA (the combination of component 1 and component 2) is not linear. The behavior of the cells will shift markedly (a nonlinear response) when substrate levels in the environment cross certain thresholds. The two biggest sources of nonlinearity in our model system are binary growth/no-growth behavior of *Bt* in the presence or absence of O_2_ and the binary capacity of *Kp* to use carbon from dextran (through its breakdown into oligosaccharides by *Bt*) in the presence or absence of *Bt*. To model the binary growth/no-growth behavior of *Bt* in the presence of O_2_, we included a conditional operator before the FBA simulations for each time step: If the O_2_ levels are above 350 nM, then the growth rate of *Bt* is set to zero and the FBA is not run for *Bt*, whereas if O_2_ levels are lower than 350 nM O_2_, then the FBA simulation for *Bt* is allowed to proceed. The value for the O_2_ growth threshold for the model is arbitrarily chosen from the concentration range reported for the aerotolerant *Bt* (*51*).

To computationally identify the regions of stability with respect to glucose and oxygen (Fig. 3C and fig. S4), we varied oxygen input flow rates at constant input glucose concentration for each glucose condition examined. We evaluated 11 glucose conditions ranging from 1 to 6 mM. For each given glucose input concentration, we started with oxygen at an input flow rate of 6 ml/min and ran the simulation for 50 hours to ensure that the system reached a steady state. We then decreased the oxygen input by intervals of 0.5 ml/min down to 0.5 ml/min, for each oxygen condition, ensuring that the system reached a steady state [we refer to the oxygen variations at 6 to 0.5 ml/min for a given constant glucose input concentration as the “forward simulations”]. The concentration of oxygen input at which *Bt* starts to grow is identified as the tipping point to the monostable *Kp-Bt* state. After running the oxygen simulation at 0.5ml/min, we increased the concentration back to 6 ml/min at intervals of 0.5 ml/min [we refer to the oxygen variations at 0.5 to 6 ml/min as the “reverse simulations”). The concentration of oxygen at which *Bt* can no longer grow and gets washed out is identified as tipping point to the monostable *Kp-*only state. The region between these two tipping points to the monostable *Kp-Bt* state in the forward simulations and the monostable *Kp*-only state in the reverse simulations is identified as the region of bistability. The colors in fig. S4 represent the steady-state concentration of *Kp* in the reverse simulations divided by the steady-state concentration of *Kp* in the forward simulations. In regions of monostability, the concentration of *Kp* is similar in both the forward and reverse simulations and therefore has a value of approximately 1.

### Continuous culture of *Kp* and *Bt*

Continuous culture experiments were carried out in a 500-ml bioreactor (miniBio Applikon Biotechnology, Delft, Netherlands) with a total culture volume of 200 ml. Minimal medium [KH_2_PO_4_ (3.85 g/liter), K_2_HPO_4_ (12.48 g/liter), (NH_4_)_2_SO_4_ (1.125 g/liter), 1× methyl methanesulfonate (20× methyl methanesulfonate: NaCl, 17.6 g/liter; CaCl_2_, 0.4 g/liter; MgCl_2_ × 6H_2_O, 0.4 g/liter; MnCl_2_ × 4H_2_O, 0.2 g/liter; and CoCl_2_ × 6H_2_O, 0.2 g/liter), Wolfe’s mineral solution (10 ml/liter) (*52*), Wolfe’s vitamin solution (10 ml/liter) (*52*), 4.17 μM FeSO_4_ × 7H_2_0, 0.25 mM cysteine, 1 μM menadione, 2 μM resazurin, dextran (1 g/liter; Sigma, D5376; average molecular weight, 1.5 × 10^6^ to 2 × 10^6^), and glucose at varying concentrations] was purged with 100% N_2,_ stored under anaerobic conditions before use, and maintained under N_2_ during operation of the CSTR. We calibrated the dissolved oxygen probe by aerating the reactor with CO_2_ (5 ml/min) and N_2_ (45 ml/min). The stable measurement without O_2_ input was taken to be 0 μM dissolved oxygen. We then sparged the reactor with O_2_ (1.7 ml/min), CO_2_ (5 ml/min), and N_2_ (43.3 ml/min). This stable measurement was taken to be 30.714 μM O_2_ [calculated assuming dissolved oxygen (6.056 mg/liter) at 1 atm and 37°C].

For the experiments, the bioreactor was sparged with total gas at 50 ml/min [O_2_ (1.7 ml/min), CO_2_ (5 ml/min), and balance of N_2_] and agitated with two six-bladed Rushton turbines operated at 750 rpm. Temperature was maintained at 37°C, and a residence time of 5 hours (input and output flow rates of 40 ml/hour) was used for all experiments. Dissolved oxygen, pH, and biomass were monitored throughout. For initial inoculation of *Kp*, 1 ml of OD 1 culture was injected through the septum and grown in batch culture until stationary phase (indicated by a leveled biomass readout and an increase of dissolved oxygen levels) before beginning continuous culture. For every experimental condition examined (input glucose concentration) in the CSTR, we waited for the system to first reach steady state (at least 24 hours). To assess whether a steady state had been reached, we monitored the total biomass in the reactor using a real-time OD probe. Once the system reached steady state, we took three samples over the course of 24 to 48 hours. The time interval between each sample collection was at least one residence time (5 hours). Residence time is defined as the time it takes to entirely exchange the volume of the reactor. For introduction of *Bt*, a log phase (OD 0.6 to 0.8) anaerobic culture grown in minimal media with 0.5% dextran and 2 mM cysteine was pelleted (5 min at 3500*g*) and washed twice using dextran/glucose-free anaerobic minimal media. Cells were carbon-starved at 37°C for 30 min, washed (once), and resuspended in dextran/glucose-free minimal media to OD 1. We used 1 ml of this *Bt* cell suspension for inoculation into the reactor, and a sample was collected immediately after inoculation. A subsequent sample was collected for quantification after at least two residence times had passed. In the *Kp*-only state conditions (0.25, 1, and 2 mM glucose), *Bt* is washed out, as described in Results. To ensure reproducibility of a washout for these conditions, the *Bt* inoculation and sample collection process were repeated a total of three times. In *Kp-Bt* state conditions (5, 2, 1, 0.25, and 0 mM glucose), where *Bt* growth persisted, reinoculation of *Bt* was no longer necessary for each new glucose steady-state condition; three samples separated by at least one residence time were collected for each steady-state condition. To collect samples, ~0.5 ml of culture was removed from the bioreactor in a 3-ml Luer-lock syringe and discarded before collection of 1.5 to 2 ml of culture. Supernatant from 700 μl of the collected sample was stored at −80°C for short-chain fatty acid analysis; a 50-μl subsample was treated with deoxyribonuclease [DNase; 2.5 μl of New England Biolabs (NEB) DNase I, 2000 U/ml per 50 μl] for subsequent DNA extraction, and two 250-μl aliquots were used for extraction of RNA.

### Quantification of bacterial abundance

CSTR culture samples were treated with NEB DNase I (final concentration, 100 U/ml) for 10 min at 37°C immediately after collection. DNA was extracted using the ZyGEM prepGEM Bacteria Kit (ZyGEM, Southampton, England) according to the manufacturer’s protocol. Samples were extracted in 100-μl total volume (20-μl culture sample and 80-μl of extraction mixture); incubated at 37°C for 15 min, 75°C for 5 min, and 95°C for 5 min; and then cooled to 4°C. DNA was stabilized by adding 10× Tris-EDTA buffer to a final concentration of 1× TE before storage at 4°C.

#### qPCR quantification

Extracted DNA was quantified by qPCR using the Eco Real-Time PCR System (Illumina, San Diego, CA, USA). The components in the qPCR mix used in this study were as follows: 1 μl of extracted DNA, 1× SsoFast EvaGreen Supermix (Bio-Rad Laboratories, Hercules, CA, USA), 500 nM forward primer, and 500 nM reverse primer. For detection of each bacterial species in the community, primer sets specific to *Bt* (forward primer, 5′-GGAGTTTTACTTTGAATGGAC-3′ and reverse primer, 5′-CTGCCCTTTTACAATGGG-3′) and *Kp* (forward primer, 5′-ATTTGAAGAGGTTGCAAACGAT-3′ and reverse primer, 5′-TTCACTCTGAAGTTTTCTTGTGTT-3′) were used. Quantification of cell concentrations were determined using DNA standards of single species prepared using 10× serial dilutions of log phase cultures extracted as above. Cell concentrations of standards were determined by hemocytometer. For conversion of OD and cell concentration to biomass concentration (gram of cell dry weight per liter), 100 ml of culture for each individual species incubated anaerobically at 37°C was harvested and pellets were dried at 80°C for ~48 hours before recording mass.

#### dPCR quantification

Archived DNA samples from the CSTR were quantified by dPCR using a QX200 Droplet dPCR (ddPCR) System (Bio-Rad). The components in the dPCR mix were as follows: 1 μl of dilutions of extracted DNA, 1× QX200 ddPCR EvaGreen Supermix (Bio-Rad), 500 nM forward primer, and 500 nM reverse primer. For detection of each bacterial species in the community, primer sets specific to *Bt* (forward primer, 5′-GGTGTCGGCTTAAGTGCCAT-3′ and reverse primer, 5′-CGGAYGTAAGGGCCGTGC-3′) and *Kp* (forward primer, 5′-ATGGCTGTCGTCAGCTCGT-3′ and reverse primer, 5′-CCTACTTCTTTTGCAACCCACTC-3′) were used. The dPCR mix was loaded into DG8 Cartridges (Bio-Rad), which were filled with QX200 Droplet Generation Oil for EvaGreen (Bio-Rad) and loaded into the QX200 Droplet Generator (Bio-Rad). The generated droplets were transferred to the C1000 Touch Thermal Cycler (Bio-Rad) for the following protocol: 5 min at 95°C, 40 cycles of 30 s at 95°C, 30 s at 60°C (*Kp*) or 65°C (*Bt*), and 30 s at 72°C (ramping rate reduced to 2°C/s) and final dye stabilization steps of 5 min at 4°C and 5 min at 90°C. The stabilized plates were loaded into the QX200 Droplet Reader and analyzed using the QuantaSoft Analysis Software (Bio-Rad).

### RNA sequencing and analysis

From the CSTR samples, a 250-μl aliquot was used for metatranscriptomic analysis. The freshly collected CSTR sample was immediately placed into Qiagen RNAprotect Bacteria Reagent (Qiagen, Hilden, Germany) for RNA stabilization. RNA was extracted using the Enzymatic Lysis of Bacteria protocol of the Qiagen RNeasy Mini Kit and processed according to the manufacturer’s protocol. DNA digestion was performed during extraction using the Qiagen RNase-Free DNase Set. The quality of extracted RNA was measured using an Agilent 2200 TapeStation (Agilent, Santa Clara, CA, USA). Extracted RNA samples were prepared for sequencing using the NEBNext Ultra RNA Library Prep Kit for Illumina (NEB, Ipswich, MA, USA) and the NEBNext Multiplex Oligos for Illumina. Libraries were sequenced at 100 single base pair reads and a sequencing depth of 10 million reads on an Illumina HiSeq 2500 System (Illumina, San Diego, CA, USA) at the Millard and Muriel Jacobs Genetics and Genomics Laboratory, California Institute of Technology. Raw reads from the sequenced libraries were subjected to quality control to filter out low-quality reads and trim the adaptor sequences using Trimmomatic (version 0.35). Because our samples were a mixture of *Kp* and *Bt* cells, to separate the reads for each species, we did the following: Reads that aligned to ribosomal RNA and transfer RNA of *Bt* and *Kp* were first removed, as those sequences contain overlapping reads between the two species. Each sample was then separately aligned to *Bt* VPI-5482 (genome accession number: GCA_000011065.1) and *Kp* MGH-78578 (genome accession number: GCA_000016305.1) using Bowtie2 (version 2.2.5) and quantified using the Subread package (version 1.5.0-p1). Gene expression was defined in transcripts per million for each species, and gene expression analysis was performed using DESeq2 (version 1.22.2; default settings, which provides two-tailored *P* values).

To determine the most differentially regulated metabolic pathway between the *Kp-Bt* and *Kp*-only states, we used an approach by Patil and Nielsen (*42*), which combines gene expression data with topological information collected from genome-scale metabolic models. Briefly, in this approach, the metabolic network is presented as a bipartite undirected graph, where metabolites and enzymes are represented as nodes (this graph is obtained from genome-scale models). Differential data can be mapped on the enzyme nodes of the graph with specification of the significance of differential gene expression for each enzyme, *i*. We used DESeq to perform our differential gene expression analysis between sample points in the two states (*Kp*-only and *Kp-Bt*) to obtain *P* values. The *P* values are subsequently converted to *Z* score for an enzyme node using the inverse normal cumulative distribution. Last, the *Z* score of each metabolite node is calculated on the basis of the normalized transcriptional response of its *k* neighboring enzymes

$$Z_{\text{metabolite}}=\frac{1}{\sqrt{k}}\sum_{1}^{k}Z_{i}$$

The metabolites with the highest *Z* scores mark the pathways with substantial regulation between two states.

## Supplementary Material

aba0353_SM.pdf

## SUPPLEMENTARY MATERIALS

Supplementary material for this article is available at http://advances.sciencemag.org/cgi/content/full/6/33/eaba0353/DC1

## Appendix

View/request a protocol for this paper from *Bio-protocol*.
